# The GGLEAM Study: Understanding Glaucoma in the Ohio Amish

**DOI:** 10.3390/ijerph18041551

**Published:** 2021-02-06

**Authors:** Andrea R. Waksmunski, Yeunjoo E. Song, Tyler G. Kinzy, Reneé A. Laux, Jane Sewell, Denise Fuzzell, Sarada Fuzzell, Sherri Miller, Janey L. Wiggs, Louis R. Pasquale, Jonathan M. Skarie, Jonathan L. Haines, Jessica N. Cooke Bailey

**Affiliations:** 1 Department of Population and Quantitative Health Sciences, Case Western Reserve University, Cleveland, OH 44106, USA; axw360@case.edu (A.R.W.); yeunjoo.song@case.edu (Y.E.S.); tgk18@case.edu (T.G.K.); ral119@case.edu (R.A.L.); jls288@case.edu (J.S.); mxf300@case.edu (D.F.); slf74@case.edu (S.F.); sdm120@case.edu (S.M.); jskarie23@gmail.com (J.M.S.); jlh213@case.edu (J.L.H.); 2 Cleveland Institute for Computational Biology, Case Western Reserve University, Cleveland, OH 44106, USA; 3 Department of Ophthalmology, Harvard Medical School, Massachusetts Eye and Ear Infirmary, Boston, MA 02114, USA; janey_wiggs@meei.harvard.edu; 4 Department of Ophthalmology, Icahn School of Medicine at Mount Sinai, New York, NY 10029, USA; louis.pasquale@gmail.com; 5 Ohio Eye Associates, Mansfield, OH 44906, USA

**Keywords:** glaucoma, vision, endophenotype, family history, founder population, Amish

## Abstract

Glaucoma leads to millions of cases of visual impairment and blindness around the world. Its susceptibility is shaped by both environmental and genetic risk factors. Although over 120 risk loci have been identified for glaucoma, a large portion of its heritability is still unexplained. Here we describe the foundation of the Genetics of GLaucoma Evaluation in the AMish (GGLEAM) study to investigate the genetic architecture of glaucoma in the Ohio Amish, which exhibits lower genetic and environmental heterogeneity compared to the general population. To date, we have enrolled 81 Amish individuals in our study from Holmes County, Ohio. As a part of our enrollment process, 62 GGLEAM study participants (42 glaucoma-affected and 20 unaffected individuals) received comprehensive eye examinations and glaucoma evaluations. Using the data from the Anabaptist Genealogy Database, we found that 80 of the GGLEAM study participants were related to one another through a large, multigenerational pedigree containing 1586 people. We plan to integrate the health and kinship data obtained for the GGLEAM study to interrogate glaucoma genetics and pathophysiology in this unique population.

## 1. Introduction

Vision loss is a significant public health concern that is worsening with the increasing size of the elderly population [1]. Glaucoma is a leading cause of irreversible blindness with about 64 million individuals affected around the world [2]. This complex phenotype is considered a collection of disorders characterized by the progressive loss of peripheral vision resulting from degeneration of the optic nerve [3]. While there are early-onset forms of glaucoma, the most common type of glaucoma, primary open-angle glaucoma (POAG), manifests in adulthood and has a complex pattern of inheritance [4]. POAG is a multifactorial condition with both genetic and environmental risk factors [5,6,7]. Genome-wide association studies of large cohorts of unrelated individuals have identified 127 loci associated with POAG risk as well as many loci associated with quantitative POAG traits called endophenotypes like intraocular pressure (IOP) and optic disc parameters [8,9,10,11,12,13,14,15,16]. However, genetic variation in these loci only accounts for less than 10% of POAG heritability [8,16]. Therefore, a substantial portion of POAG heritability remains unexplained [17].

Previous studies have shown the utility of working with founder populations and population isolates to elucidate genetic variation for glaucoma [18,19,20,21,22,23,24,25] and other age-related ocular traits [26,27,28,29]. The Amish comprise an isolated, founder population that is culturally and genetically segregated from the general population of European descent [30]. They typically practice a conservative, uniform lifestyle that includes similar dietary habits, occupations, and physical activity as well as minimal smoking [31]. Nearly all present-day Amish are descendants of a few hundred Swiss–German Anabaptists who emigrated to the United States to escape religious persecution in Europe in the late eighteenth and early nineteenth centuries [32,33]. The resettlement of these individuals in North America resulted in a population bottleneck that has been sustained across generations because Amish typically marry within their faith group and non-Amish individuals rarely join the Amish community [34]. Therefore, the Amish are a valued population in genetics research due to their relatively homogeneous environments and their reduced genetic variation due to their founder event and consanguinity [35].

Our multidisciplinary team set out to establish the Genetics of GLaucoma Evaluation in the AMish (GGLEAM) study to understand the prevalence and risk factors of glaucoma in Amish communities in Ohio. This represents a previously unexplored area in genetics research, especially given that the incidence of common, age-related forms of glaucoma are unknown in the Amish. Furthermore, we hypothesize that studying a complex ocular disease like glaucoma in a genetically and environmentally homogeneous population, like the Amish, will facilitate the discovery of novel loci associated with glaucoma and its endophenotypes and aid in our understanding of its pathophysiology. This work also highlights the importance of establishing a working relationship with study participants in biomedical research, especially those from special populations.

## 2. Materials and Methods

### 2.1. Study Participants

The participants in the Genetics of GLaucoma Evaluation in the AMish (GGLEAM) study are Amish individuals living in and around Holmes County, Ohio. The Holmes County, Ohio Amish community comprises one of the largest Amish settlements in North America [36]. It began in 1809 when Amish settlers moved there from Somerset County, Pennsylvania [37]. Our initial contacts with this community were made over twenty years ago through advertising in the local Amish papers and meeting with community leaders to establish a working relationship with the Amish community in Holmes County as previously described [38,39,40,41,42,43,44,45,46]. Due to their lifestyle, Amish abstain from using modern technology in their homes [30,33]; therefore, study participants were recruited for this study using door-to-door methods and through advertisements in local newspapers. The recruitment for our GGLEAM study branched from the ongoing Amish Eye Study, which recruited Amish individuals from Ohio, Indiana, and Pennsylvania to interrogate age-related macular degeneration (AMD) genetics and identify possible biomarkers for AMD from optical coherence tomography (OCT) [46]. In that study, some participants self-reported prior glaucoma diagnoses, and we found that all the self-reported glaucoma diagnoses were clinically confirmed (positive predictive value: 100%). These observations inspired us to increase our engagement with the Amish in Holmes County, Ohio and launch the GGLEAM study to focus on glaucoma in this population.

The GGLEAM study enrollment process was comprised of two visits with participants. In the first visit, the study coordinator consented the study participants in their homes, and study participants completed health and family medical history questionnaires (Supplementary Materials), which were based on the questionnaires developed for the Collaborative Amish Aging and Memory Project (NIH grants AG058066 and AG019085) and the Amish Eye Study (NIH grant EY023164). The second visit occurred at the Ohio Eye Associates office in Mansfield, Ohio at which the participant received a comprehensive eye examination performed by a fellowship-trained MD glaucoma specialist. During this visit, a blood sample was also drawn by the study coordinator for DNA extraction and biomarker analysis. The examination included visual acuity assessment by Snellen eye chart, automated 24-2 Humphrey visual field testing (Zeiss, Oberkochen, Germany), stereo optic disc photography, optical coherence tomography assessment of the optic disc, retinal nerve fiber layer and ganglion cell complex (Cirrus SD-OCT, Zeiss, Oberkochen, Germany), gonioscopy, central corneal thickness measurement by Pachmate 2 (DGH Technology, Inc., Exton, PA, USA), assessment of corneal hysteresis via the Ocular Response Analyzer (Reichert Technologies, Buffalo, NY, USA), intraocular pressure (IOP) measurement with Goldman applanation tonometry, detailed slit lamp biomicroscopy, and fundus exam via indirect ophthalmoscopy following pupil dilation. Glaucoma diagnoses were defined by current American Academy of Ophthalmology Preferred Practice Guidelines [47,48]. Briefly, glaucoma determination was based on IOP measurements taking into account corneal thickness and hysteresis as well as optic nerve examination, visual field testing, and assessment of the retinal nerve fiber layer and ganglion cell layer complex.

Individuals were invited to participate in the study if they met one of the following criteria: (i) reported to have or were diagnosed with any type of glaucoma; (ii) were at least 40 years old and did not have glaucoma; or (iii) had family members with glaucoma. Individuals under 40 without a prior diagnosis of glaucoma or family history of glaucoma were not invited to participate in the study. All study participants provided informed consent, and the study was conducted within the guidelines of the Declaration of Helsinki. The study protocol was approved by the institutional review board at Case Western Reserve University (IRB-2017-2067).

### 2.2. Amish Pedigree

The Amish are a culturally isolated population with extensive genealogical records dating back to their emigration to North America [49]. Community-based directories and research-based resources, described elsewhere, have been developed to curate these data [50]. To determine the relatedness of the GGLEAM study participants, we queried the Anabaptist Genealogy Database (AGDB) [51]. Based on these pedigree data, we constructed an all-connecting path pedigree (ACP) to depict all known familial relationships among study participants and their ancestors. The ACP was drawn using the Pedigraph software tool [52]. Kinship and inbreeding coefficients were calculated using KinInbcoef software and genealogical information from the all-connecting path pedigree [53].

## 3. Results

### 3.1. GGLEAM Study Participants

Thus far, we have enrolled 81 study participants from the Amish community in and near Holmes County, Ohio. Of these individuals, we have obtained phenotypic data from comprehensive eye exams and health questionnaires for 62 individuals to date. A fellowship-trained MD glaucoma specialist determined glaucoma type and severity based on the results of each individual’s comprehensive glaucoma examination and testing results. Most of the glaucoma-affected study participants (88%) have primary open-angle glaucoma (POAG) (Table 1). On average, individuals with glaucoma were older than individuals without glaucoma (67.6 years old and 56.95 years, respectively) (Table 1).

### 3.2. Family History of Glaucoma

Family history is a well-established risk factor for glaucoma [4,54,55,56]. Individuals who have first-degree relatives with glaucoma have a 10-fold higher risk of developing glaucoma compared to individuals without glaucoma-affected first-degree relatives [57]. We enrolled study participants based on their self-reported or clinically confirmed diagnosis of glaucoma as well as their self-reported family history of glaucoma. Specifically, we asked study participants if they had a family history of glaucoma in first-degree relatives or extended family members. As a consequence of the interrelatedness and close-knit nature of this population, several of the study participants were from the same Amish nuclear families. We found that about 66% of the GGLEAM study participants had first-degree relatives with glaucoma (Table 2). Of the 42 glaucoma-affected Amish individuals in our study, 80.95% had first-degree relatives affected by glaucoma, and 69.05% had extended family members with glaucoma (Table 2). Most of the unaffected individuals in this study reported that they did not have a family history of glaucoma (Table 2).

### 3.3. Quantitative Ocular Measurements

The majority of the GGLEAM study participants (*n* = 62) underwent comprehensive eye exams that yielded various quantitative ocular measures for 42 glaucoma-affected individuals and 20 unaffected individuals (Table 3). This includes quantitative ocular measurements for 37 POAG-affected individuals (Table 4). Sixteen of the glaucoma-affected study participants either received IOP lowering medications (i.e., prostaglandin analogs, beta-blockers, alpha agonists, oral carbonic anhydrase inhibitors, topical carbonic anhydrase inhibitors, or cholinergic agents) or had prior surgery (i.e., glaucoma filtering surgery, laser iridotomy, argon laser trabeculoplasty, selective laser trabeculoplasty, or minimally invasive glaucoma surgery). Average IOP was higher among individuals with glaucoma (i.e., average IOP: 15 mmHg) compared to individuals without glaucoma (average IOP: 14 mmHg) (Table 3). The average IOP in study participants with POAG was about 15 mmHg (Table 4). The average vertical cup-to-disc ratio (VCDR) was over 1.6 times higher in glaucoma-affected individuals (OD VCDR: 0.62 ± 0.12, OS VCDR: 0.66 ± 0.12) compared to unaffected individuals (OD VCDR: 0.38 ± 0.15; OS VCDR: 0.37 ± 0.16) (Table 3). The average VCDR in the right and left eyes of POAG-affected individuals were 0.63 and 0.66, respectively (Table 4). Although the average refractive error for individuals without glaucoma was substantially lower than for study participants with glaucoma, the ranges of values observed for both groups overlapped (Table 3). The mean central corneal thickness (CCT) was lower in glaucoma-affected individuals compared to unaffected individuals (Table 3). The average CCT for POAG-affected individuals was also lower than the average CCT observed in unaffected individuals (Table 3 and Table 4). Average axial length was nearly the same in glaucoma-affected individuals compared to unaffected individuals (Table 3 and Table 4).

### 3.4. Genealogy of GGLEAM Study Participants

**Figure 1:**
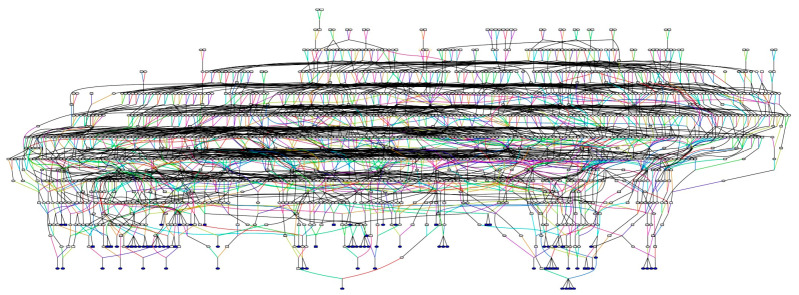
All-connecting path pedigree of GGLEAM study participants. This multigenerational pedigree connects 80 of the 81 Amish individuals enrolled in the GGLEAM study and includes 1586 individuals. Genealogy data was obtained from the Anabaptist Genealogy Database (AGDB) [43]. The 80 GGLEAM study participants are highlighted in blue. Men are represented by squares, and females are represented by circles. Pedigree was drawn using the Pedigraph software tool.

The Amish represent a unique population in genetics research due to their recent founding event and the extensive genealogical records available for this community [32,35,51,58]. One of these resources includes the Anabaptist Genealogy Database (AGDB) [51,59]. We queried the AGDB and found that 80 of the 81 GGLEAM study participants are related to one another through a 1586-person all-connecting path (ACP) pedigree (Figure 1). One individual enrolled in this study only had parental information in the AGDB and could not be connected to the pedigree. Kinship coefficients were calculated among the 80 connected GGLEAM study participants using KinInbcoef [53]. The average kinship coefficient among these study participants was 0.023 (SEM: 0.00064). The maximum kinship coefficient observed was 0.273, and the minimum was 0.00257. The average inbreeding coefficient calculated by KinInbcoef was 0.014 (SEM: 0.000747).

## 4. Discussion

Glaucoma is a leading cause of irreversible blindness, but its genetic architecture and disease etiology are not fully understood. We established the GGLEAM study to understand glaucoma risk in the Ohio Amish, which are a population isolate. While previous studies in the Amish examined congenital glaucoma [60] or described glaucoma as a clinical feature in a few individuals with a homozygous mutation in the *SAMHD1* gene associated with cerebral vasculopathy and early onset stroke [61], glaucoma risk and prevalence have not been extensively studied in the Amish population. Therefore, this study can provide the foundation for future work assessing glaucoma risk in this population, which could inform understanding of glaucoma risk in general. To date, we have enrolled 81 Amish individuals from in and around Holmes County, Ohio, and 62 of these study participants have received comprehensive eye exams. Moving forward, we plan to enroll additional Ohio Amish community members in our study and obtain data from glaucoma-specific eye exams for all of the GGLEAM study participants.

Because our study design includes obtaining glaucoma status and quantitative eye measurements of the GGLEAM study participants, we can study the genetic variation potentially associated with glaucoma and its endophenotypes. Examining heritable endophenotypes such as IOP and VCDR rather than disease status alone increases the statistical power to detect associated loci for a heterogeneous phenotype like glaucoma [18]. Identifying genetic variants associated with these glaucoma-related traits may improve our understanding of glaucoma development and disease pathophysiology [62]. Additionally, studying a complex trait like glaucoma in an isolated, founder population like the Amish may allow for the identification of novel genetic variants that have been indiscernible from previous genetics studies in large datasets of unrelated individuals due to low minor allele frequency (MAF) [17]. In traditional case-control genome-wide association studies, the sample size needed to detect trait-associated variants increases with 1/MAF [17]. Therefore, sample sizes in these population-based cohorts are in the tens of thousands. By comparison, association studies of rare variants in families require lower sample sizes if disease-associated variants are present since they are likely to be inherited by other family members [63]. This aspect of family-based study designs is augmented in studies with population isolates, which can have densely affected families with potentially causal variants inherited identical-by-descent from a small set of common ancestors [34,64]. The Amish resettlement in North America and generations of intra-faith marriages has resulted in the accumulation of alleles that are rare in the general population of European descent.

The all-connecting path pedigree we constructed for the GGLEAM study participants using data from the AGDB [51] confirmed that we are effectively working with one large family, which will greatly aid in future genetics studies of this cohort. Furthermore, the average kinship and inbreeding coefficients we calculated for these individuals were 0.023 and 0.014, respectively. The estimated inbreeding coefficient for the Amish population, in general, is 0.0151 [65]. The average kinship coefficient previously calculated for Amish families in Ohio and Indiana was 0.019 [41]. Leveraging kinship information enables us to account for familial relationships among study participants, which are highly interconnected due to generations of endogamy in the population [35]. We can then utilize this information in family-based association tests between glaucoma affection status and SNP genotype to identify novel glaucoma risk variants or in gene-set methods (i.e., kernel, burden, and collapsing methods) to identify rare variants for glaucoma [17,66,67,68].

In addition to being genetically homogeneous as a result of their recent population bottleneck and generations of intra-faith marriages, the Amish adhere to a conservative lifestyle that is mostly consistent across Amish communities, which are predominantly in rural areas [35]. Therefore, environmental exposures, modifiable health behaviors, and lifestyle factors are more homogeneous in the Ohio Amish compared to the general population of European descent. These features of the Amish population make them a valued population to investigate both genetic and environmental risk factors for glaucoma as well as the possible interactions among these risk factors. Through health, physical activity, environmental exposure, and family history questionnaires in the GGLEAM study (Supplementary Materials), we aim to investigate non-genetic factors pertaining to glaucoma risk in this population. We are especially interested in factors that have been inconsistently associated with glaucoma risk and development, including diet, physical activity, caffeine consumption, smoking, and alcohol consumption [69,70,71]. As a part of their cultural traditions, the Amish generally do not use motorized vehicles; therefore, they have higher activity levels than non-Amish individuals [30,72]. Most Amish community members consume a similar diet that consists of foods that are rich in carbohydrates and lipids as well as homegrown fruits and vegetables [73]. They also typically abstain from smoking and consuming alcoholic beverages [73,74]. All 62 GGLEAM study participants who completed our questionnaires stated that they had never smoked, and only one individual self-reported alcohol consumption. Therefore, our GGLEAM study is well-positioned to assess the contributions of risk factors like physical activity, diet, and caffeine consumption in a uniquely homogeneous population that generally prohibits smoking and alcohol consumption. Increased knowledge of the effects and interplay of genetic and environmental risk factors may broaden our understanding of glaucoma pathophysiology, facilitate earlier disease detection based on more comprehensive risk profiles, and inspire the development of novel therapeutics to treat glaucoma.

Amish communities in North America have been engaged in genetics research since the 1960s [35,58]. These early efforts led to better understanding of various Mendelian genetic disorders [33], and several studies have also shown the utility of studying complex traits in Amish families due to their reduced heterogeneity in genetic variation and environmental exposures [17,26,28,29,38,39,40,41,42,43,44,45,46,75,76,77,78,79,80,81,82,83,84,85,86,87,88,89,90,91,92,93,94,95]. Initial research efforts with Amish communities involved collaboration among researchers and local Amish liaisons who were familiar with the families in the community, could speak Pennsylvania Dutch, and understood the customs and values of the Amish [58]. Some of these practices were continued as more research teams began working with the Amish population, including the Amish Research Program [96,97,98] and the Amish Eye Study [46].

The factors most valued by the Amish include faith, family, and community [36,58]. Their participation in research has partially been shaped by their altruistic nature and their belief that, by participating in these studies, they are helping others [97]. To ensure that our study design and enrollment practices were respectful of these values, we engaged in door-to-door recruitment methods, performed study enrollments in the study participants’ homes, and used paper questionnaires to generate some of our study data. Our study coordinators also built rapport with the GGLEAM study participants from engagement in prior research studies and years of community involvement [26,46]. With the growing field of genomics research in diverse and understudied populations, it is paramount that researchers form longitudinal partnerships with study participants, especially those from vulnerable populations [99,100].

## 5. Conclusions

Glaucoma significantly contributes to global cases of vision loss and blindness. While large population-based cohorts have successfully identified numerous loci contributing to glaucoma risk, most of the additive genetic variance for glaucoma is not attributable to known loci. We started the GGLEAM study to study glaucoma risk in the Amish population by ascertaining individuals with and without glaucoma from in and around Holmes County, Ohio. To date, 81 Amish individuals have been enrolled in this study, and phenotypic data has been generated for 62 of these individuals through health, lifestyle, environmental exposure, and family history questionnaires as well as comprehensive eye exams. We determined that these study participants are highly interrelated through a multigenerational pedigree and plan to incorporate this kinship information into our future genetics studies. Our hope is that the health, phenotypic, and genetic data we are gathering, together with the extensive genealogical records for this special population, will be invaluable in expanding our understanding of the genetic epidemiology of glaucoma.

## Figures and Tables

**Table 1:** Features of Genetics of GLaucoma Evaluation in the AMish (GGLEAM) Study Participants. Of the 42 glaucoma-affected Amish in this study, 37 individuals have POAG and 5 have another form of glaucoma including 1 person with chronic angle closure glaucoma and 4 individuals with pigmentary glaucoma. Age values represent the study participants’ ages at their eye exams. The age range for study participants with glaucoma was 40–85, and the age range for unaffected individuals was 42–81.

		Affected	Unaffected
**N**		42	20
**Age**	**Mean ± SD**	67.60 ± 11.39	56.95 ± 10.33
**Sex**	**Male**	19	7
	**Female**	23	13

**Table 2:** Family history of glaucoma in GGLEAM study participants. First-degree relatives include parents and siblings. Extended family includes aunts, uncles, and cousins.

Family History of Glaucoma		Affected (%)	Unaffected (%)	All (%)
**First-Degree Relatives**	No	8 (19.05)	13 (65)	21 (33.87)
	Yes	34 (80.95)	7 (35)	41 (66.13)
**Extended Family**	Unknown	1 (2.38)	1 (5)	2 (3.23)
	No	12 (28.57)	14 (70)	26 (41.94)
	Yes	29 (69.05)	5 (25)	34 (54.84)

**Table 3:** Quantitative eye measurements. Measurements were obtained from eye exams performed at the Ohio Eye Associates office. These data were obtained for 42 glaucoma-affected study participants and 20 unaffected individuals. IOP: intraocular pressure. VCDR: vertical cup-to-disc ratio. OD: oculus dexter (right eye). OS: oculus sinister (left eye). Refractive error: Spherical equivalent from distance refraction. D: diopters.

	Affected	Unaffected
	Average ± SD (Range)	Average ± SD (Range)
**OD IOP** (mmHg)	15.1 ± 4.0 (3–22)	13.5 ± 2.8 (8–19)
**OS IOP** (mmHg)	15.4 ± 3.7 (9–22)	13.8 ± 3.1 (8–21)
**OD VCDR**	0.62 ± 0.12 (0.30–0.90)	0.38 ± 0.15 (0.10–0.64)
**OS VCDR**	0.66 ± 0.12 (0.40–0.90)	0.37 ± 0.16 (0.06–0.62)
**OD Refractive error** (D)	0.59 ± 1.55 (−4.00, +4.50)	−0.17 ± 2.34 (−6.00, +3.25)
**OS Refractive error** (D)	0.49 ± 1.54 (−3.00, +5.00)	−0.21 ± 2.32 (−6.50, +3.00)
**OD CCT** (Microns)	549 ± 43 (440–658)	559 ± 29 (516–606)
**OS CCT** (Microns)	547 ± 42 (466–638)	564 ± 34 (514–627)
**OD Axial Length** * (mm)	23.70 ± 0.86 (22.02–25.47)	23.71 ± 1.13 (21.58–26.30)
**OS Axial Length** * (mm)	23.60 ± 0.85 (21.86–25.20)	23.58 ± 1.10 (21.59–25.98)

* These values were calculated based on measurements from 33 glaucoma-affected individuals and 20 controls.

**Table 4:** Quantitative eye measurements for POAG-affected GGLEAM study participants. Measurements were obtained from eye exams performed at the Ohio Eye Associates office for 37 POAG-affected individuals. IOP: intraocular pressure. VCDR: vertical cup-to-disc ratio. OD: oculus dexter (right eye). OS: oculus sinister (left eye). Refractive error: Spherical equivalent from distance refraction. D: diopters.

	POAG
	Average ± SD (Range)
**OD IOP** (mmHg)	15.0 ± 4.2 (3–22)
**OS IOP** (mmHg)	15.1 ± 3.6 (9–22)
**OD VCDR**	0.63 ± 0.11 (0.30–0.85)
**OS VCDR**	0.66 ± 0.11 (0.40–0.90)
**OD Refractive error** (D)	0.56 ± 1.59 (−4.00, +4.50)
**OS Refractive error** (D)	0.53 ± 1.57 (−3.00, +5.00)
**OD CCT** (Microns)	547 ± 43 (440–658)
**OS CCT** (Microns)	545 ± 42 (466–638)
**OD Axial Length** * (mm)	23.66 ± 0.88 (22.02–25.47)
**OS Axial Length** * (mm)	23.55 ± 0.84 (21.86–25.09)

* These values were calculated based on measurements from 29 individuals with POAG.

## Data Availability

Deidentified data are, in principle, available to qualified investigators once institutional agreements have been reached.

## Supplementary Materials

The following are available online at https://www.mdpi.com/1660-4601/18/4/1551/s1, Supplementary Item 1: GGLEAM study questionnaire for family history of eye disease, Supplementary Item 2: Questionnaire for GGLEAM study participants’ physical activity and environmental exposure, and Supplementary Item 3: Questionnaire for GGLEAM study participants’ health and activities.
