# Supplementary material for: The GGLEAM Study: Understanding Glaucoma in the Ohio Amish
Source: Int J Environ Res Public Health. 2021 Feb 6;18(4):1551. doi: 10.3390/ijerph18041551 (PMC7915874; doi:10.3390/ijerph18041551)
Supplement: Supplementary file 1 [file ijerph-18-01551-s001.pdf]

Supplementary Item 1. GGLEAM study questionnaire for family history of eye disease.

1) Please indicate if your mother or father has been diagnosed with any of the following eye disorders in one or both eyes:

**Eye Disorder**	**Mother**	**Father**
**AMD:**	____ Yes ___ No ___ Don't know	____ Yes ___ No ___ Don't know
**Glaucoma:**	____ Yes ___ No ___ Don't know	____ Yes ___ No ___ Don't know
**Cataracts:**	____ Yes ___ No ___ Don't know	____ Yes ___ No ___ Don't know
**Other causes of blindness:**	____ Yes ___ No ___ Don't know	____ Yes ___ No ___ Don't know

If YES, please specify: ____

2) Please enter the number of brothers, sisters, and/or children that have been diagnosed with any of the following eye disorders in one or both eyes (enter "0" if none):

**Eye Disorder**	**Brother(s)**	**Sister(s)**	**Child(ren)**
**AMD:**	# ____ Don't know ____	# ____ Don't know ____	# ____ Don't know ____
**Glaucoma:**	# ____ Don't know ____	# ____ Don't know ____	# ____ Don't know ____
**Cataracts:**	# ____ Don't know ____	# ____ Don't know ____	# ____ Don't know ____
**Other causes of blindness:**	# ____ Don't know ____	# ____ Don't know ____	# ____ Don't know ____

Please specify: ____

3) Please enter the number of other blood relatives that have been diagnosed with any of the following eye disorders in one or both eyes (enter "0" if none):

**Eye Disorder**	**Other Blood Relatives**
**AMD:**	# _____ Specify: ________________________________ Don't know _____
**Glaucoma:**	# _____ Specify: ________________________________ Don't know _____
**Cataracts:**	# _____ Specify: ________________________________ Don't know _____
**Other causes of blindness:**	# _____ Specify: ________________________________ Don't know _____

Supplementary Item 2. Questionnaire for GGLEAM study participants' physical activity and environmental exposure.

1) In the past week, how many days did you participate in physical activity or exercise that was moderate, hard, or very hard? This includes walking continuously, brisk walking that makes you breathe harder than normal, doing heavy housework such as mopping or scrubbing, and heavy yard/garden work.

_______ # of days _______ Don't Know

2) On those days when you participated in some physical activity or exercise, for how many minutes were you active? That is, on average, how many minutes each day were you active?

_______ Average Daily Minutes _______ Don't Know

3) What is the source of your drinking water?

_____ Well or Spring

_____ Provided by City/Town

_____ Purchased from Store or Water Supply Company

4) Have you ever been or are you currently exposed to any chemical fertilizers and/or pesticides at home, in the garden, on the farm, or at work?

_____ Yes _____ No

If yes, please provide details here (type of chemical, length and frequency of exposure, etc.):

____

____

5) How many caffeinated drinks do you consume on a daily basis?

____ 1-2 ____ 3-4 ____ 5 or more

Type (circle): Coffee Tea Soft Drinks

Supplementary Item 3. Questionnaire for GGLEAM study participants' health and activities.

1) Have you smoked at least 100 cigarettes or used smokeless tobacco in your lifetime?

○ Yes ○ No ○ Don't Know

If YES:
1a) Did you ever smoke cigarettes or use smokeless tobacco (snuff) at least once per week?

○ Yes ○ No ○ Don't Know

If YES:
1b) What year (or age) did you start?

Year (or [ ][ ][ ][ ] Age) ○ [ ][ ] Don't Know

1c) Do you use any tobacco products now?

○ Yes ○ No ○ Don't Know

If NO: 1d) What year (or age) did you last use tobacco products at least once per week?

Year [ ][ ][ ][ ] (or Age) ○ [ ][ ] Don't Know

1e) On average, how many cigarettes do (did) you smoke per day? (1 pack=20) How much smokeless tobacco? (1 can, ½ can, etc.)

[ ][ ] or _____ cans ○ Don't Know

2) Have you ever taken a multivitamin at least once a week for a month or more?

○ Yes ○ No ○ Don't Know

If YES:
2a) What year did you start?

Year [ ][ ][ ][ ] (or Age) ○ [ ][ ] Don't Know

2b) Are you currently taking a multivitamin?

○ Yes ○ No ○ Don't Know

If NO: 2c) What year did you stop?

Year [ ][ ][ ][ ] (or Age) [ ][ ] ○ Don't Know

2d) How many tablets do (did) you take per week?

[ ][ ] ○ Don't Know

2e) What brand do you usually use?

______________________________ ○ Don't Know

FOR WOMEN ONLY (FOR MEN, PLEASE SKIP THIS SECTION)

3) How old were you when you started having menstrual periods?

AGE: [ ][ ] ○ Don't Know ○ Never had a menstrual period

*** If you have never had a menstrual period, end exam here ***

4) Have you ever been diagnosed with gestational diabetes?

○ Yes ○ No ○ Gravida 0

If YES:

4a) In how many pregnancies did you develop gestational diabetes?

[ ] out of [ ]

5) Have you ever been diagnosed with pre-eclampsia?

○ Yes ○ No ○ Gravida 0

If YES:

5a) In how many pregnancies did you develop pre-eclampsia?

[ ] out of [ ]

FOR WOMEN ONLY (CONTINUED)

6) Have you reached menopause (the change of life)?

○ Yes ○ No ○ Don't Know

If YES:

4a) At what age did your menstrual periods stop?

AGE: [ ][ ] ○ Don't Know

4b) Why did they stop?

- ○ Natural menopause (change of life)
- ○ Total hysterectomy (uterus and both ovaries removed)
- ○ Hysterectomy without ovaries removed
- ○ Partial hysterectomy with one ovary left
- ○ Radiation or chemotherapy
- ○ Other (please specify): ____
- ○ Don't know ____________________________________

7) Have you ever used hormone pills, shots, creams, or patches to help regulate menstrual periods, premenstrual symptoms (PMS) or hot flashes, or prevent bone loss caused by menopause (the change of life)? Brand names for these hormones include Alora, Climara, Estratest, Estring, Fempatch, Ortho Dienestrol, Premphase, Prempro, Tace, Vivelle, Estraderm, Estrace, Estinyl, Estratab, Menest, Premarin, Provera, Ogen, Ortho-Est, and Estrovis.

○ Yes ○ No ○ Don't Know

If NO, end of exam. If yes, continue to next page.

If YES:

7a) What year did you start?

Year [ ][ ][ ][ ] (or Age) [ ][ ] ○ Don't Know

7b) Are you currently taking hormones? ○ Yes ○ No ○ Don't Know

If NO: 7c) What year did you stop?

Year [ ][ ][ ][ ] (or Age) [ ][ ] ○ Don't Know

8) You may have started and stopped taking hormones several times. How long OVERALL have you taken hormones?

[ ][ ] Year(s) [ ][ ] Month(s) ○ Don't Know
